# Multicenter phase II study of biweekly CAPIRI plus bevacizumab as second-line therapy in patients with metastatic colorectal cancer (JSWOG-C3 study)

**DOI:** 10.1007/s10147-019-01473-3

**Published:** 2019-05-29

**Authors:** Nobuaki Suzuki, Shoichi Hazama, Takeshi Nagasaka, Hiroaki Tanioka, Yasuo Iwamoto, Yuji Negoro, Masami Yamauchi, Michiya Kobayashi, Hiroshi Okuda, Noriaki Fujishima, Taku Nishimura, Naoki Yamanaka, Kazuhiro Toyota, Yoshiko Mori, Yuki Nakagami, Mototsugu Shimokawa, Hiroaki Nagano, Masazumi Okajima

**Affiliations:** 1 Department of Gastroenterological, Breast and Endocrine Surgery, Yamaguchi University Graduate School of Medicine, 1-1-1 Minami-Kogushi, Ube, 755-8505 Japan; 2 Department of Translational Research and Developmental Therapeutics Against Cancer, Yamaguchi University School of Medicine, Ube, Japan; 3 Department of Clinical Oncology, Kawasaki Medical School, Kurashiki, Japan; 4 Department of Medical Oncology, Hiroshima City Hiroshima Citizens Hospital, Hiroshima, Japan; 5 Department of Medical Oncology, Kochi Health Sciences Center, Kochi, Japan; 6 Division of Clinical Oncology, Hiroshima Prefectural Hospital, Hiroshima, Japan; 7 Cancer Treatment Center, Kochi Medical School Hospital, Kochi, Japan; 8 Surgery and Endoscopic Surgery, Onomichi General Hospital, Onomichi, Japan; 9 Department of Surgery, Fukuda Clinic of Internal Medicine, Heart and Digestive, Kochi, Japan; 10 Department of Surgery, Kokura Memorial Hospital, Kokura, Japan; 11 Department of Surgery, Japanese Red Cross Yamaguchi Hospital, Yamaguchi, Japan; 12 Department of Surgery, National Hospital Organization Higashihiroshima Medical Center, Hiroshima, Japan; 13 Department of Gastroenterological Surgery, Okayama University Graduate School of Medicine, Dentistry and Pharmaceutical Sciences, Okayama, Japan; 14 Cancer Biostatistics Laboratory, Clinical Research Institute, National Kyushu Cancer Center, Fukuoka, Japan; 15 Department of Surgery, Hiroshima City Hiroshima Citizens Hospital, Hiroshima, Japan

**Keywords:** Bevacizumab, CAPIRI, Biweekly, Second line, mCRC

## Abstract

**Background:**

Triweekly capecitabine plus irinotecan (CAPIRI) was not a replacement for fluorouracil, leucovorin, and irinotecan (FOLFIRI) in the treatment of metastatic colorectal cancer (mCRC) because of the potential for greater toxicity. Recently, it has reported that mCAPIRI is well tolerated and non-inferior to FOLFIRI. In this study, we conducted a multicenter phase II trial to assess the efficacy and safety of biweekly CAPIRI plus bevacizumab as second-line chemotherapy for mCRC with reduced toxicity and preserved efficacy.

**Methods:**

Patients with mCRC who had received prior chemotherapy, including oxaliplatin-based regimens, were eligible for this study. The treatment protocol administered capecitabine at 1000 mg/m^2^ twice daily from the evening of day 1 to the morning of day 8, intravenous irinotecan at 150 mg/m^2^ on day 1, and bevacizumab at 10 mg/kg on day 1 every 2 weeks. Primary endpoints for this study were progression-free survival (PFS) and safety. Secondary endpoints were overall survival (OS), time to treatment failure, response rate (RR), and disease control rate (DCR).

**Results:**

Fifty-one patients were enrolled in this study. Median PFS was 5.5 months [95% confidence interval (CI) 4.23–7.40 months], and median OS was 13.5 months (95% CI 11.57–20.23 months). The RR was 14.6% (95% CI 6.5–28.4%), and the DCR was 66.7% (95% CI 51.5–79.2%). Hypertension was the most common Grade 3 adverse event (27.5%), followed by neutropenia (17.6%). Only two patients suffered from grade 3 hand–foot syndrome.

**Conclusions:**

In mCRC patients, biweekly CAPIRI + bevacizumab appears effective and feasible as a second-line chemotherapy with relatively low toxicities, and has potential as a useful substitute for FOLFIRI + bevacizumab.

## Introduction

Colorectal cancer (CRC) is one of the leading causes of cancer-related deaths and the most common cancer type, with more than one million new cases diagnosed annually worldwide [1–3]. In recent years, new regimens for colon cancer combining chemotherapy and biological agents have improved the overall survival (OS) and progression-free survival (PFS) [4–7]. Standard treatments for patients with metastatic CRC (mCRC) usually consist of combination chemotherapy based on fluorouracil or capecitabine plus either oxaliplatin or irinotecan, and a targeted agent such as bevacizumab, cetuximab, or panitumumab [8–10]. The most commonly used chemotherapy regimens are fluorouracil with folinic acid plus oxaliplatin (FOLFOX), fluorouracil with folinic acid plus irinotecan (FOLFIRI), and capecitabine plus oxaliplatin (XELOX). Particularly as a second-line regimen, triweekly capecitabine plus irinotecan (CAPIRI) was not a replacement for fluorouracil, leucovorin, and irinotecan (FOLFIRI) in the treatment of metastatic colorectal cancer (mCRC) because of the potential for greater toxicity [11–13]. However, some phase II trials have suggested that a modified reduced-dose CAPIRI regimen (irinotecan 200 mg/m^2^ on day 1 plus capecitabine 800 mg/m^2^ twice daily on days 1–14) once every 3 weeks offered favorable tolerability and efficacy in the second-line setting [14]. Recently, an Asian phase III trial, the AXEPT study has reported Mcapiri, is well tolerated and non-inferior to FOLFIRI [15]. We also found another phase I/II study of biweekly capecitabine and irinotecan plus bevacizumab as second-line chemotherapy in patients with mCRC, but that was conducted at only one high-volume center hospital [16]. However, we could not find any multicenter trials for biweekly CAPIRI plus bevacizumab (10 mg/kg) in a second-line setting. From the perspective of patient care and scheduled administration of chemotherapy, some advantages are seen in a biweekly regimen, which is easier to manage than a weekly or triweekly regimen, but the usefulness of this method is still unclear. Therefore, given the current situation in Japan in which oxaliplatin-based chemotherapy is provided as the primary therapy, we have envisioned a multicenter phase II study to assess the efficacy and safety of biweekly CAPIRI plus bevacizumab (10 mg/kg) as second-line chemotherapy for mCRC. In relation to a biweekly regimen for capecitabine, we have experience with achieving acceptable toxicity and good efficacy in a trial for another CAPEOX regimen using a biweekly capecitabine regimen (capecitabine at 1000 mg/m^2^ twice a day on days 1–7) [17]. Since the key drug in the second-line therapy is considered to be irinotecan, a CAPIRI regimen that can be administered every other week using irinotecan at 150 mg/m^2^ as the standard dose in Japan with an oral fluoropyrimidine is important. In this study, we, therefore, conducted a multicenter phase II trial to assess the efficacy and safety of biweekly CAPIRI plus bevacizumab as second-line chemotherapy for mCRC with reduced toxicity and preserved efficacy.

## Patients and methods

### Study design

This single-arm, phase II, multi-institutional clinical trial was conducted to determine the efficacy and safety of a combination regimen of bevacizumab (10 mg/kg) with biweekly CAPIRI as second-line chemotherapy in patients with mCRC. The study was performed in accordance with the Declaration of Helsinki and the ethical guidelines for clinical studies. The study protocol was approved by the institutional review board of Yamaguchi University (H23-182), and was then started after approval from the relevant institutional review boards at each of the participating institutions. Trial registration: this study has been registered in the University Hospital Medical Information Network (UMIN) Clinical Trials Registry as UMIN 000009280.

### Inclusion criteria

Eligibility criteria for patients were as follows: histologically proven colorectal 5 adenocarcinoma; unresectable or recurrent disease; a measurable lesion confirmed 28 days prior to enrollment; prior chemotherapy for metastatic or recurrent disease, including oxaliplatin-based regimens with bevacizumab; preserved organ functions [neutrophil count ≥ 1.5 × 10^3^/mm^3^; platelets ≥ 10.0 × 10^3^/mm^3^; hemoglobin ≥ 9.0 g/dl; total bilirubin ≤ 1.5 mg/dl; AST/ALT ≤ 100 IU/l; creatinine ≤ 1.5 mg/dl; urinary protein ≤ 1 + ]; Eastern Cooperative Oncology Group performance status (ECOG PS) 0–1; expected survival ≥ 3 months; age ≥ 20 years; no double cancer; and provision of written, informed consent to participate. On the other hand, the dose adjustment based on the measurement result of UGT1A1 gene polymorphism before the first dose administration is not indispensable.

### Exclusion criteria

Exclusion criteria for patients were as follows: severe peritoneal ascites or pleural effusion; jaundice; intestinal obstruction; severe renal failure; brain tumor and brain metastasis recognized on imaging; duplicated cancers with a disease-free period of less than 5 years; radiation therapy carried out within 4 weeks before registration; antithrombotic agent administered to thrombosis within 10 days before registration; unhealed traumatic fracture; uncontrollable hypertension; history of myocardial infarction within 1 year before registration; and patients judged otherwise inappropriate by a doctor.

### Treatment plan

Treatment consisted of irinotecan at 150 mg/m^2^ as an intravenous infusion on day 1 every 2 weeks, capecitabine at 1000 mg/m^2^ twice daily on days 2–8, followed by 1 week of rest, and bevacizumab at 10 mg/kg as an intravenous infusion on day 1 every 2 weeks.

Treatment was continued until one of the following occurred: progressive disease, treatment was not resumed even after 28 days from the last administration, administration difficulty due to severe adverse effects, or decision to stop treatment at the discretion of the treating physician. If chemotherapy was delayed, the administration of bevacizumab was also delayed. If irinotecan or bevacizumab was discontinued, capecitabine and irinotecan or capecitabine and bevacizumab were to be continued unless unacceptable toxicity was observed. If capecitabine was interrupted beyond 28 days, treatment could not be continued.

### Assessment

Tumors were evaluated using computed tomography before initiation of treatment (within 2 weeks), at 4 weeks after initial treatment, and at 6-week intervals (allowance ± 2 weeks) thereafter. The primary end points of the present study were PFS and safety. The result of complete response or partial response was confirmed after a subsequent minimum of 4 weeks. Secondary end points included OS, time to treatment failure (TTF), response rate (RR), and disease control rate (DCR). RR and DCR assessed by investigators according to RECIST version 1.1 criteria. No independent radiological review committee was established. Adverse events were monitored and graded according to the National Cancer Institute Common Terminology Criteria for Adverse Events version 4.0.

### Statistical analyses

This single-arm phase II study was designed to assess the PFS and safety of a combination regimen of bevacizumab with biweekly CAPIRI as second-line chemotherapy in patients with mCRC. In the previous report, the median PFS with CAPIRI as second-line chemotherapy was 5.1 months [18]. The target sample size of 50 (43 eligible patients and 10% ineligible patients) was based on expected and threshold PFS of 8.0 and 5.1 months, respectively, with *α* = 0.05 and *β* = 0.1.

Secondary endpoints were OS, TTF, RR, and DCR. Efficacy analyses were performed on the intention-to-treat population, i.e., patients who received at least one course of study medication. Safety analyses were performed on patients who received study medication at least once. Distributions of PFS, OS, and TTF times were estimated using the Kaplan–Meier method. Qualitative variables were described using absolute and relative frequencies. Quantitative variables were described with means, medians, and standard deviation (SD). All analyses were performed using R language (version 3.5.1).

## Results

### Baseline characteristics

A total of 52 patients were enrolled in the study at 13 Japanese centers between January 2013 and July 2015. One patient failed the screening process and did not meet the entry criteria, and so could not be treated within the study regimen. The remaining 51 patients received treatment and were included in the intention-to-treat and safety populations. Patient characteristics are summarized in Table 1. Median patient age was 66 years (range 41–82 years), and patients comprised 29 males (56.9%) and 22 females (43.1%). ECOG PS was 0 in 42 patients (82.4%) and 1 in 9 patients (17.6%). The most common primary tumor site was the colon in 31 patients (60.8%). The most common histology of the primary tumor was moderately differentiated adenocarcinoma in 33 patients (64.7%). The most common site of metastasis was multiple sites in 29 patients (56.9%), followed by metastasis limited to the liver in 13 patients (25.5%), and limited to another single region in 9 patients (17.6%). UGT1A1 status was wild type in 31 patients (59.6%), *6 heterozygote in 13 patients (25%), *28 heterozygote in six patients (11.5%), compound heterozygote in one patient (1.9%), and unmeasured in one patient (1.9%). 16 (31.4%) had received the previous therapy with cetuximab or panitumumab, and 31 patients (60.8%) had received the previous therapy with bevacizumab.

**Table 1:** Patient characteristics (*N* = 51)

	*N*	%
Median age (range), years	66 (41–82)	
Sex		
Male	29	56.9
Female	22	43.1
ECOG PS		
PS = 0	42	82.4
PS = 1	9	17.6
Site of primary tumor		
Colon	31	60.8
Rectum	18	35.3
Cecum	2	3.9
Histology of primary tumor		
Well differentiated adenocarcinoma	11	21.6
Moderately differentiated adenocarcinoma	33	64.7
Poorly differentiated adenocarcinoma	3	5.9
Others	4	7.8
Metastatic sites		
Liver limited	13	25.5
Other single region	9	17.6
Multiple site	29	56.9
UGT1A1 status		
Wild	11	21.6
*6 Polymorphism	7	13.7
*28 Polymorphism	4	7.8
Compound heterozygote	0	0
Unmeasured	15	29.4
Previous therapy		
Previous therapy with panitumumab or cetuximab	16	31.4
Previous therapy with bevacizumab	31	60.8

*ECOG* Eastern Cooperative Oncology Group, *PS* performance status

### Efficacy

**Fig. 1:**
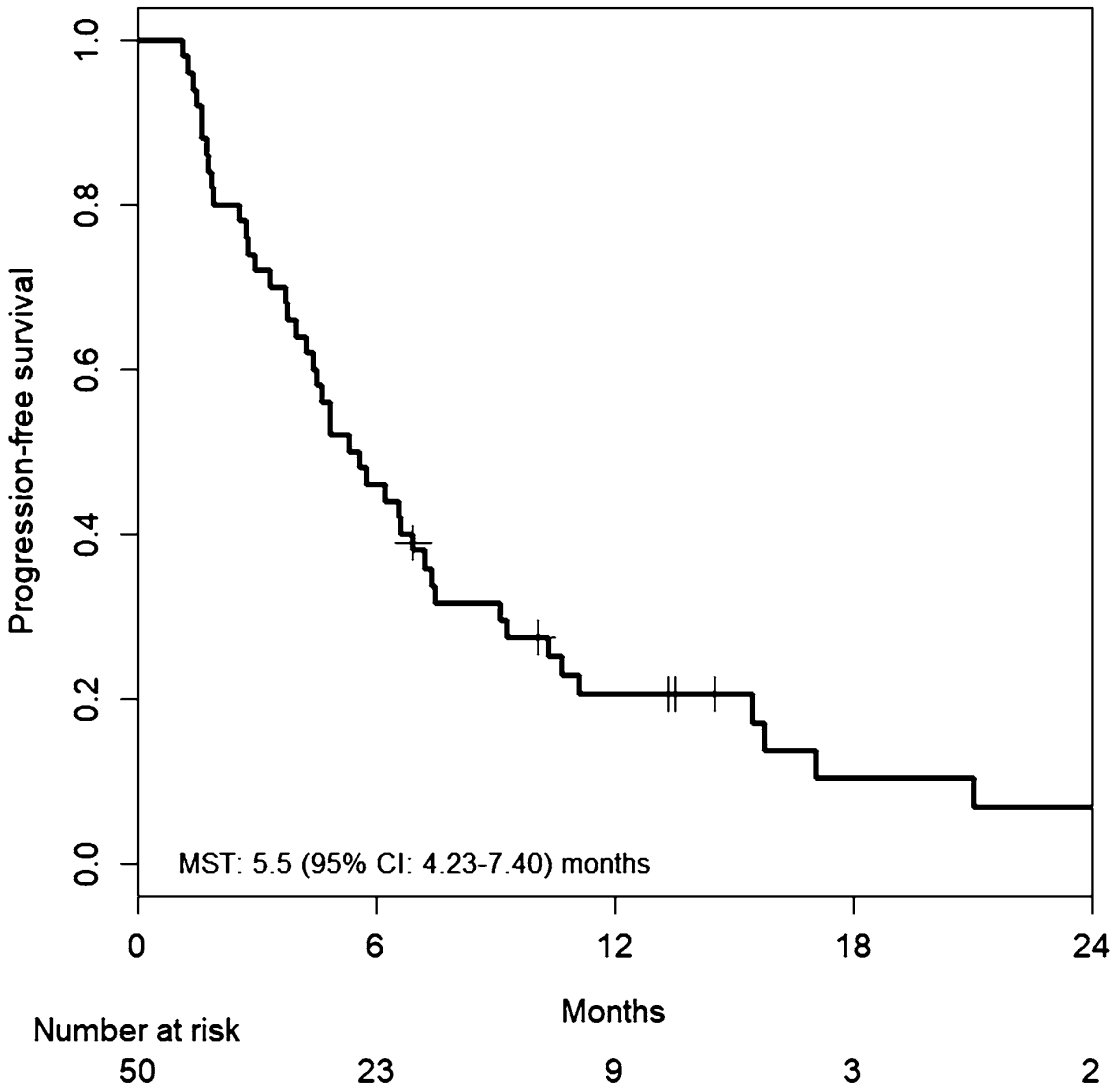
Kaplan–Meier estimates of progression-free survival (PFS) in the full analysis set of metastatic colorectal cancer (mCRC) patients treated with biweekly CAPIRI + bevacizumab as second-line treatment. Median PFS was 5.5 months (95% CI 4.23–7.40 months)

**Fig. 2:**
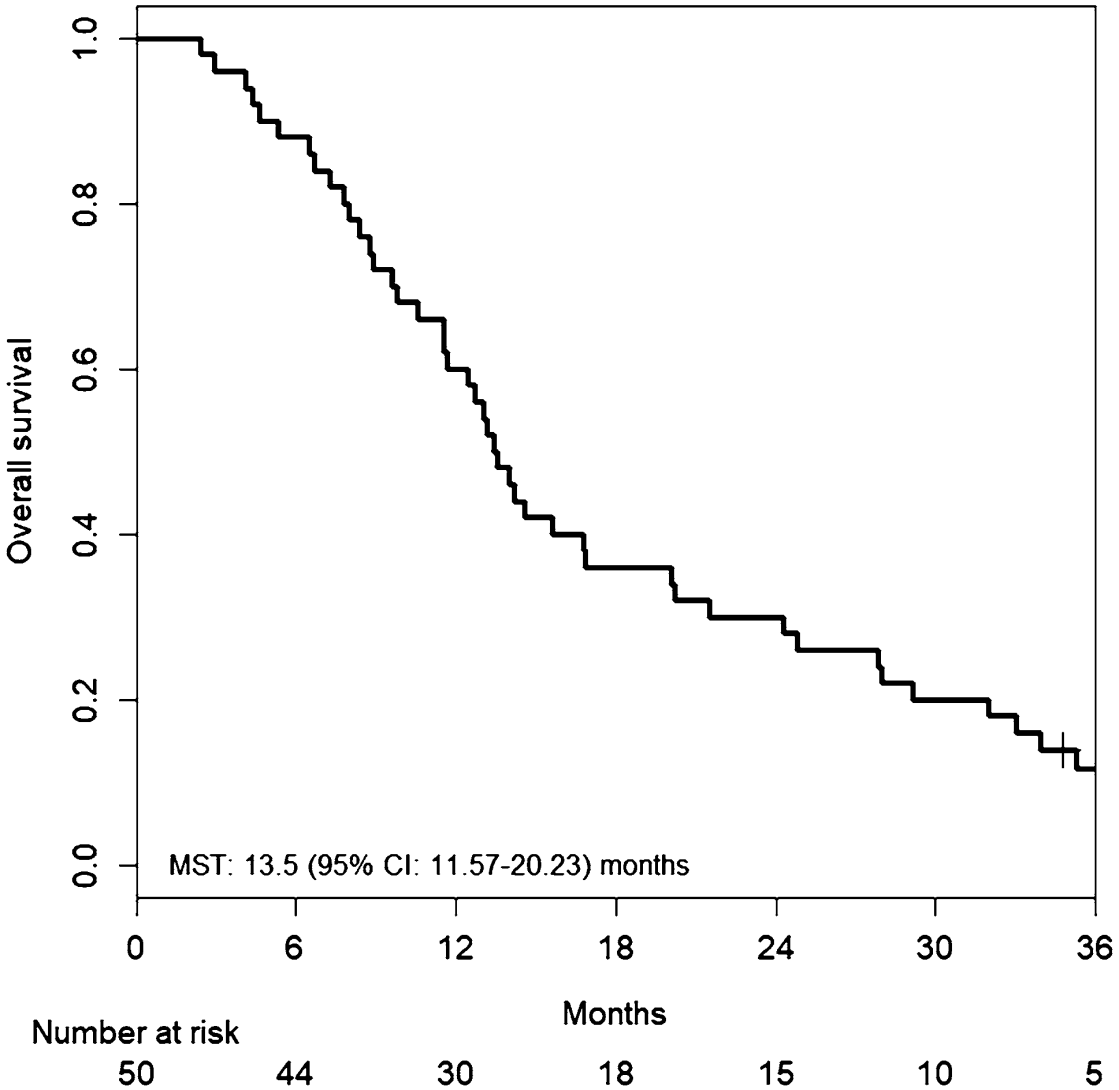
Kaplan–Meier estimates of overall survival (OS) in the full analysis set of mCRC patients treated with biweekly CAPIRI + bevacizumab as second-line treatment. Median OS was 13.5 months (95% CI 11.57–20.23 months)

**Fig. 3:**
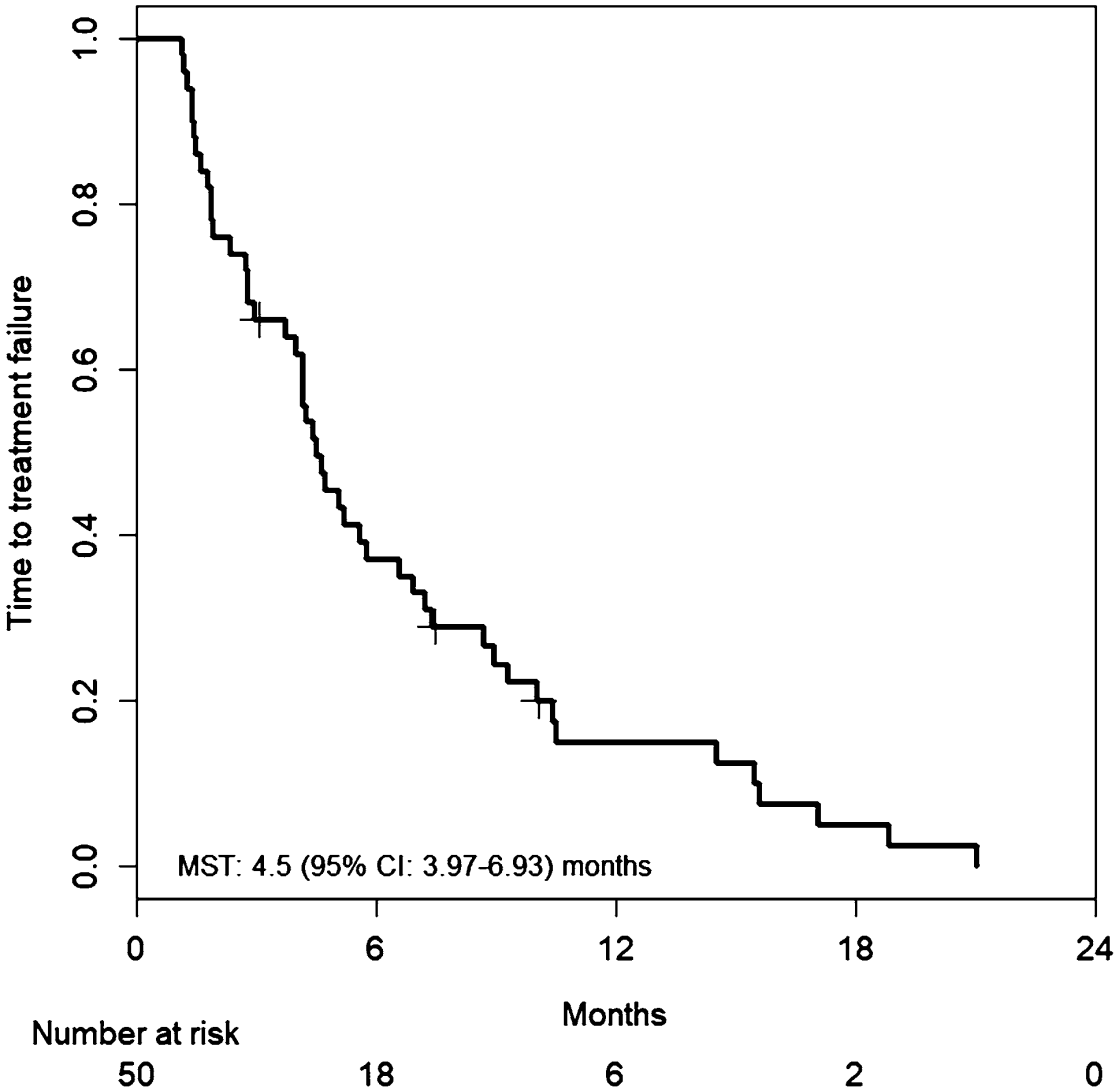
Kaplan–Meier estimates of time to treatment failure (TTF) in the full analysis set of mCRC patients treated with biweekly CAPIRI + bevacizumab as second-line treatment. Median TTF was 4.5 months (95% CI 3.97–6.93 months)

**Fig. 4:**
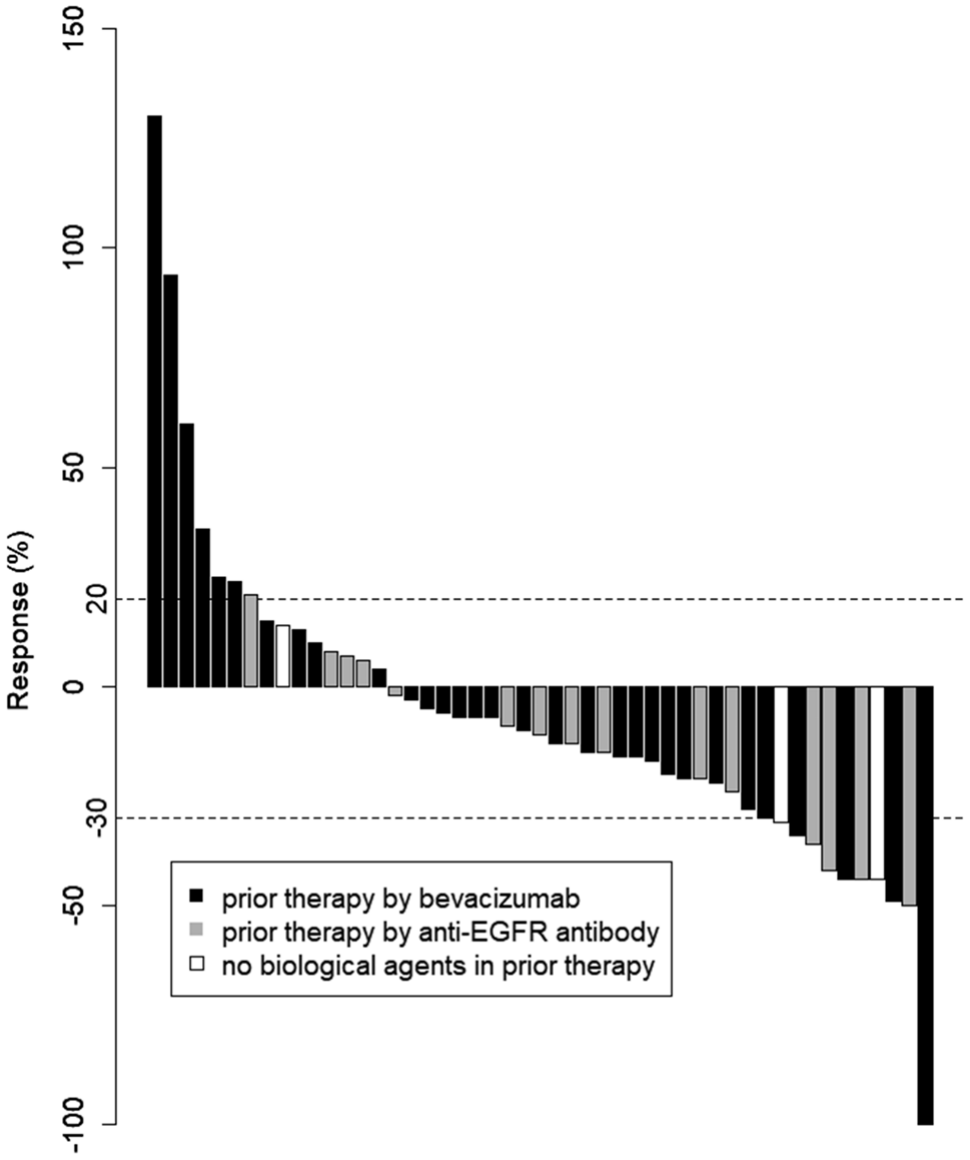
Waterfall-plot analysis of maximum target lesion response compared to the baseline in patients with mCRC treated with second-line biweekly CAPIRI plus bevacizumab (*N* = 49). Apart from one patient administered anti-EGFR antibody, the other six patients who had target lesions increased over 20% used bevacizumab as first-line therapy. *CAPIRI* capecitabine plus irinotecan regimen

**Fig. 5:**
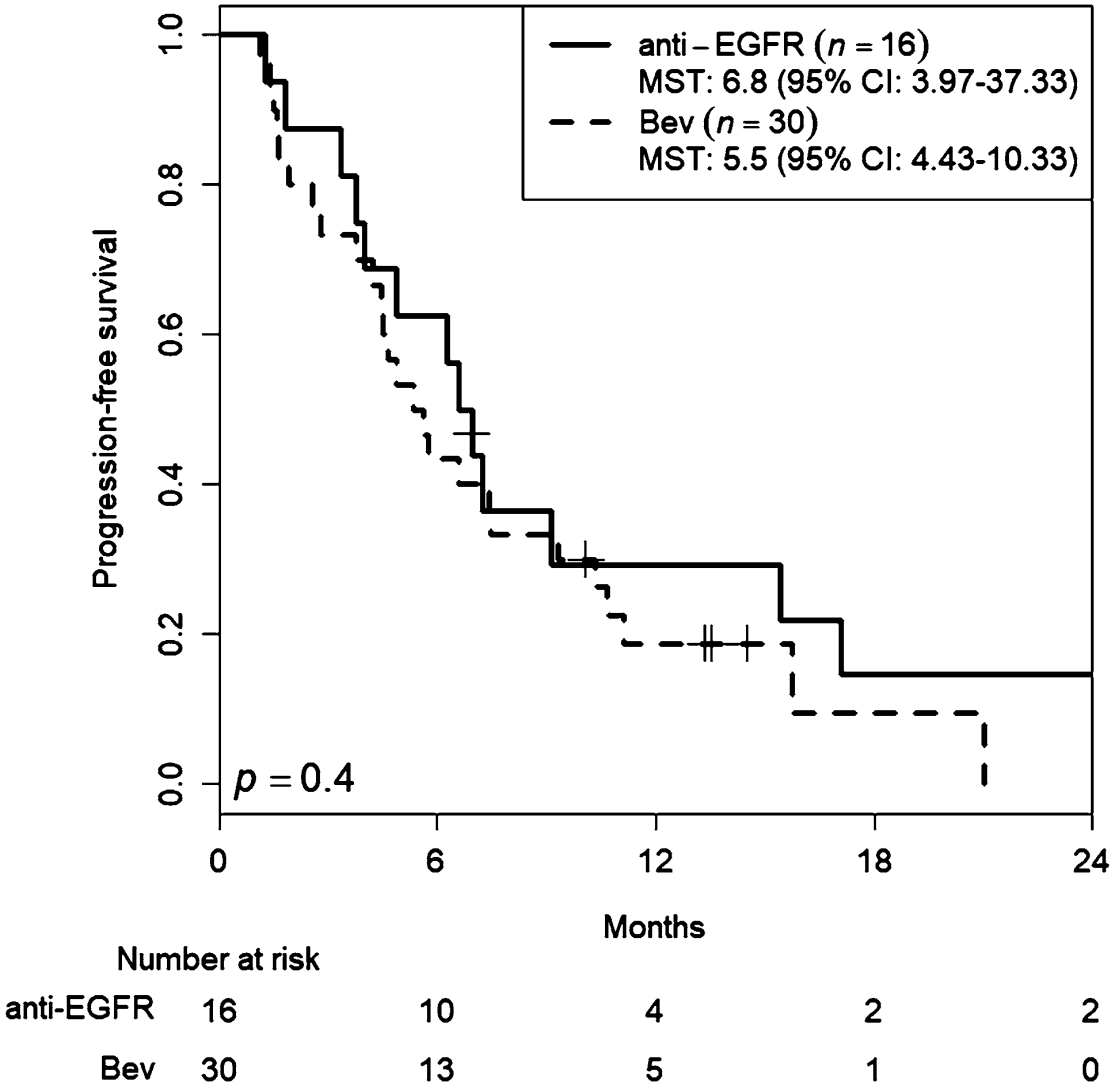
Kaplan–Meier estimates of progression-free survival (PFS) in the full analysis set of metastatic colorectal cancer (mCRC) patients treated with bevacizumab versus anti-EGFR (cetuximab or panitumumab) as first-line treatment. Median PFS was 5.5 months (95% CI 4.43–10.33) and 6.8 months (95% CI 3.97–37.33) for the bevacizumab group and anti-EGFR group, respectively. *Bev* bevacizumab. *p* = 0.4, log-rank test

Tumor response was assessed in 50 patients. Patients were followed for a median of 13.5 months (range 2.4–56.1 months). Median number of treatment cycles was 8.0 (range 1–42 treatment cycles). Median cumulative doses of each agent were: 1780 mg/m^2^/day (range 621–2151 mg/m^2^/day) for capecitabine; 138.5 mg/m^2^/course (range 83.4–151.2 mg/m^2^/course) for irinotecan; and 10.0 mg/kg/course (range 5.2–10.6 mg/kg/course) for bevacizumab. Median relative dose intensities of each agent were 0.88 (range 0.31–1.08) for capecitabine; 0.92 (range 0.56–1.01) for irinotecan; and 1.00 (range 0.52–1.06) for bevacizumab. Treatment efficacies are summarized in Table 2. Overall, one complete and six partial responses were observed, and response rate was 14.6% (95% CI 6.5–28.4%). Because 25 patients had stable disease, the DCR was 66.7% (95% CI 51.5–79.2%). Median PFS was 5.5 months (95% CI 4.23–7.40 months) (Fig. 1). Median OS was 13.5 months (95% CI 11.57–20.23 months) (Fig. 2). Median TTF was 4.5 months (95% CI 3.97–6.93 months) (Fig. 3). Maximum target lesion response compared to baseline is shown in Fig. 4. Almost all patients (six of seven patients) who showed an increased volume of main lesions over 20% had used bevacizumab in the first-line therapy. For the sub-analysis of PFS according to first-line treatment, median PFSs were 5.5 months (95% CI 4.43–10.33) for the bevacizumab group and 6.8 months (95% CI 3.97–37.33) for the anti-EGFR (cetuximab or panitumumab) group (Fig. 5).

**Table 2:** Efficacy of treatment with XELIRI + Bevacizumab

Tumor response	Number (%)
CR	1 (2%)
PR	6 (12%)
SD	25 (50%)
PD	16 (32%)
NE	2 (4%)
RR [95% CI]	7 (14.6%) [6.5–28.4]
DCR [95% CI]	32 (66.7%) [51.5–79.2]

All patients (*N* = 50)

*XELIRI* xeloda and irinotecan, *CR* complete response, *PR* partial response, *SD* stable disease, *PD* progressive disease, *NE* not evaluated, *RR* response rate, *DCR* disease control rate, *CI* confidence interval

### Safety

With the exception of one patient who suffered from grade 4 intestinal pneumonia, all adverse events (AEs) were within grade 3 in this population. Hypertension was the most common Grade 3 adverse event (27.5%), followed by neutropenia (17.6%) (Table 3). Anorexia, nausea, vomiting, diarrhea, and hand–foot syndrome were common at low grades, but at grade 3 showed frequencies of 11.8%, 7.8%, 2.0%, 3.9%, and 3.9%, respectively. Only two patients suffered from grade 3 hand–foot syndrome. No treatment-related mortality was seen among patients in this study.

**Table 3:** Adverse events according to CTCAE version 4.0 (*N* = 51)

Adverse events	All grades, *N* (%)	grade 3, *N* (%)
Hematological		
Neutropenia	28 (55.0)	9 (17.6%)
Anemia	40 (78.4)	1 (2.0)
Thrombocytopenia	26 (51.0)	0
Febrile neutropenia	2 (3.9)	2 (3.9)
Non-hematological		
Anorexia	32 (62.7)	6 (11.8)
Nausea, vomiting	23 (45.1)	4 (7.8)
Diarrhea	24 (47.1)	1 (2.0)
Stomatitis	12 (23.5)	2 (3.9)
Hand–foot syndrome	23 (45.1)	2 (3.9)
Total bilirubin increase	6 (11.8)	0
AST increase	32 (62.7)	0
ALT increase	20 (39.2)	0
Creatinine increase	11 (21.6)	0
Hypertension	37 (72.5)	14 (27.5)
Proteinuria	24 (47.1)	0 (0.0)
Bleeding	11 (21.6)	1 (2.0)
Intestinal pneumonia	2 (3.9)	1 (2.0, grade 4)

*ALT* alanine aminotransferase, *AST* aspartate aminotransferase

## Discussion

Irinotecan is one of the key drugs for the treatment of mCRC, along with oxaliplatin [19]. These drugs are often combined with fluorouracil plus leucovorin in regimens such as FOLFIRI or FOLFOX [20, 21]. In recent years, a treatment method replacing intravenous 5-FU with oral fluorinated pyrimidine has been under development, and several methods of administration have been reported using mainly CAPIRI therapy as the first line. Weekly CAPIRI therapy [22], which is mainly administered with CPT-11: 70 mg/m^2^ every week, and CPT-triweekly CAPIRI therapy administered as 200–300 mg/m^2^ on day 1 [12]. At the time, we started this trial, no evidence was available regarding CAPIRI plus bevacizumab (10 mg/kg) as a second-line chemotherapy, especially as a biweekly regimen. Some studies have suggested that irinotecan is associated with significant gastrointestinal toxicities, and several dosages and administration regimens have been investigated to maximize efficacy and tolerability [23]. To the best of our knowledge, this study is one of the first multicenter phase II clinical trials to evaluate the clinical efficacy and safety of biweekly CAPIRI plus 10 mg/kg bevacizumab as a second-line therapy in patients with mCRC. This study demonstrated that administering capecitabine–irinotecan plus bevacizumab every 2 weeks is feasible and tolerable as a second-line treatment option for patients with mCRC. The target sample size of 50 (43 eligible patients, 10% ineligible patients) was based on expected and threshold PFSs of 8.0 and 5.1 months, respectively, with *α* = 0.05 and *β* = 0.1. The median PFS of 5.5 months met our primary endpoint, but was slightly shorter than that of reported trials investigating biweekly CAPIRI with bevacizumab treatment as a second-line chemotherapy [16]. However, this was a multicenter study involving general hospitals, with a median age of 66 years, close to the age actually clinical experienced, so our PFS results may be more practically applicable than those from a single high-volume center hospital. Adverse events in the present study were within acceptable rates. Grade 3 febrile neutropenia appeared in two patients (4%) in our study, similar to rates reported from other studies. We observed grade 3 diarrhea in only one patient (2%), much better than the 10–19% reported by others [11, 16, 23]. Apart from one patient administered anti-EGFR antibody, the other six patients who had increased target lesions by over 20% had used bevacizumab as a first-line treatment (Fig. 4). For the sub-analysis of PFS, the median PFS was 5.5 months (95% CI 4.43–10.33) and 6.8 months (95% CI 3.97–37.33) for bevacizumab group and anti-EGFR (cetuximab or panitumumab) group, respectively, as first-line treatment (Fig. 5).Treatment with biweekly CAPIRI + bevacizumab appears more effective in cases, where bevacizumab is not used in the initial treatment [24].

An Asian phase III trial, the AXEPT study was conducted in the same period as our study [15]. That study compared the efficacy and safety of the mCAPIRI regimen with that of standard FOLFIRI, with or without bevacizumab, in both regimens, as a second-line therapy for mCRC. They concluded that mCAPIRI group is well tolerated and non-inferior to FOLFIRI group in terms of OS. Median OS was 16.8 months (95% CI 15.3–19.1 months) in the mCAPIRI group. In our study, median OS was 13.5 months. The median relative dose intensity in this study was slightly better than that of the AXEPT trial. In the present study, grade 3/4 adverse events of diarrhea, neutropenia, and hand–foot syndrome were less frequent than the standard triweekly CAPIRI regimen [25, 26]. On the other hand, those were almost the same as in the mCAPIRI group in the AXEPT trial. As only two patients suffered from grade 3 hand–foot syndrome, but it was manageable. In contrast, we selected a bevacizumab dose of 10 mg/kg on expectation of greater effectiveness in this regimen. A bevacizumab dose of 10 mg/kg was effective for some cases and tolerable, but the numbers of patients who suffered from hypertension and proteinuria were increased for all grades (72.5% and 47.1%, respectively). After we finished enrolling the patients, another phase III trial was reported that compared bevacizumab at 5 mg/kg and 10 mg/kg [27]. No significant difference was seen between groups and the frequency of hypertension was increased in the 10 mg/kg group. Our present study may be feasibly continued and can be administered while maintaining quality of life (QOL) for patients. Patients with unresectable or advanced colorectal cancer sometimes have poor physical condition, such as ECOG PS 1–2. In addition, Japan is now becoming an aging society. We can, therefore, select this regimen comfortably and usefully not only for suitable patients, but for elderly and high-risk patients.

To avoid the grade 3 hypertension, we recommended bevacizumab 5 mg/kg. Based on this limitation, we concluded that biweekly CAPIRI + bevacizumab is well balanced and tolerable in terms of efficacy and safety as a second-line chemotherapy. Biweekly CAPIRI + bevacizumab (5 mg/kg) could be a replacement for FOLFIRI + bevacizumab in patients with mCRC.
